# Polyphenols-based intelligent oral barrier membranes for periodontal bone defect reconstruction

**DOI:** 10.1093/rb/rbae058

**Published:** 2024-05-28

**Authors:** Enni Chen, Tianyou Wang, Zhiyuan Sun, Zhipeng Gu, Shimeng Xiao, Yi Ding

**Affiliations:** State Key Laboratory of Oral Diseases & National Center for Stomatology & National Clinical Research Center for Oral Diseases, West China Hospital of Stomatology, Sichuan University, Chengdu 610041, China; Department of Periodontics, West China Hospital of Stomatology, Sichuan University, Chengdu 610041, China; College of Polymer Science and Engineering, State Key Laboratory of Polymer Materials Engineering, Sichuan University, Chengdu 610065, China; State Key Laboratory of Oral Diseases & National Center for Stomatology & National Clinical Research Center for Oral Diseases, West China Hospital of Stomatology, Sichuan University, Chengdu 610041, China; Department of Periodontics, West China Hospital of Stomatology, Sichuan University, Chengdu 610041, China; College of Polymer Science and Engineering, State Key Laboratory of Polymer Materials Engineering, Sichuan University, Chengdu 610065, China; State Key Laboratory of Oral Diseases & National Center for Stomatology & National Clinical Research Center for Oral Diseases, West China Hospital of Stomatology, Sichuan University, Chengdu 610041, China; Department of Periodontics, West China Hospital of Stomatology, Sichuan University, Chengdu 610041, China; State Key Laboratory of Oral Diseases & National Center for Stomatology & National Clinical Research Center for Oral Diseases, West China Hospital of Stomatology, Sichuan University, Chengdu 610041, China; Department of Periodontics, West China Hospital of Stomatology, Sichuan University, Chengdu 610041, China

**Keywords:** polyphenols, intelligent, oral barrier membrane, periodontal bone defect, reconstruction

## Abstract

Periodontitis-induced periodontal bone defects significantly impact patients’ daily lives. The guided tissue regeneration and guided bone regeneration techniques, which are based on barrier membranes, have brought hope for the regeneration of periodontal bone defects. However, traditional barrier membranes lack antimicrobial properties and cannot effectively regulate the complex oxidative stress microenvironment in periodontal bone defect areas, leading to unsatisfactory outcomes in promoting periodontal bone regeneration. To address these issues, our study selected the collagen barrier membrane as the substrate material and synthesized a novel barrier membrane (PO/4-BPBA/Mino@COL, PBMC) with an intelligent antimicrobial coating through a simple layer-by-layer assembly method, incorporating reactive oxygen species (ROS)-scavenging components, commercial dual-functional linkers and antimicrobial building blocks. Experimental results indicated that PBMC exhibited good degradability, hydrophilicity and ROS-responsiveness, allowing for the slow and controlled release of antimicrobial drugs. The outstanding antibacterial, antioxidant and biocompatibility properties of PBMC contributed to resistance to periodontal pathogen infection and regulation of the oxidative balance, while enhancing the migration and osteogenic differentiation of human periodontal ligament stem cells. Finally, using a rat periodontal bone defect model, the therapeutic effect of PBMC in promoting periodontal bone regeneration under infection conditions was confirmed. In summary, the novel barrier membranes designed in this study have significant potential for clinical application and provide a reference for the design of future periodontal regenerative functional materials.

## Graphical abstract

**Figure:**
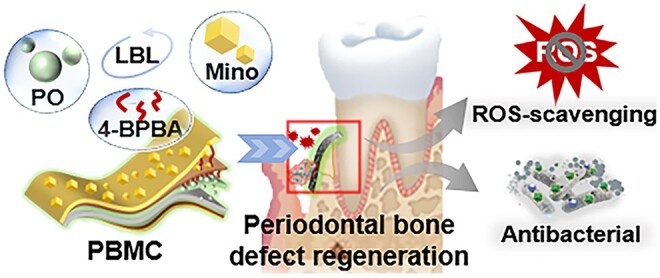

## Introduction

Alveolar bone defects secondary to periodontitis, the most common symptoms, can damage the integrity of periodontal tissue, compromising aesthetic and occlusal functions and severely impacting patients’ quality of life [1–3]. For clinicians, the primary objective of periodontal treatment is to achieve regeneration of periodontal tissue, but this still remains a major clinical challenge [4]. Guided tissue regeneration (GTR) and guided bone regeneration (GBR) techniques have emerged as promising and critical surgical strategies for the regeneration of periodontal tissue and the reconstruction of periodontal bone defects [5, 6]. Nonetheless, due to the complex oral environment, the clinical prognosis based on GTR and GBR surgical treatment is unpredictable [7]. The open environment of the oral cavity is favorable for the colonization and reproduction of various opportunistic pathogens [4, 8], and barrier membranes as implant materials are vulnerable to bacterial infection, which not only affect the surgical effect but also lead to surgical failure [9]. Moreover, in the unique immune-inflammatory microenvironment of periodontal bone defects, immune cells are excessively recruited and activated due to the long-term bacterial infection and chronic inflammatory stimulation, followed by the release of excessive reactive oxygen species (ROS), mediating oxidative stress and inducing oxidative damage, which seriously affect periodontal bone regeneration [10–13]. However, current commercialized barrier membranes predominantly function as physical barriers (preventing the ingrowth of soft tissue into the defect site) and lack appropriate antibacterial, antioxidative and osteogenic differentiation-promoting abilities. They may even trigger material-dependent inflammatory responses [14]. Consequently, it is an urgent need to develop novel barrier membranes that integrate antibacterial, antioxidative and osteogenic functionalities to address the limitations of traditional options.

Significant efforts have been invested in the development of innovative barrier membranes [15]. Novel barrier membranes with antibacterial, antioxidative and bone regeneration-promoting capabilities have been successfully constructed by combining various bioactive ingredients and advanced preparation technologies (freeze-drying method [16], evaporative phase separation method [17], electrospinning [18], solution casting forming [15] and 3D printing technology [19]). Despite their promise, the preparation methods of these novel barrier membranes are complex, posing a risk of early burst release of loaded drugs, and exhibiting limitations in terms of key properties toward periodontal bone defects. The layer-by-layer (LBL) technique, a straightforward and effective material modification approach, involves the sequential assembly of materials on substrate materials using electrostatic forces, hydrogen bonding and other intermolecular interactions, which allows the facile incorporation of multiple synthons, enhancing their functionality [20, 21].

Minocycline (Mino) is a semi-synthetic tetracycline with broad-spectrum antibacterial effects that has been recognized by the US food and drug administration (FDA) as the most effective agent approved for the treatment of bacterial infection [22–24], and has been widely used in the clinical treatment of periodontitis. Beyond its primary antibacterial role, previous studies have indicated that Mino can enhance osteoblast activation, stimulate the production of osteogenic proteins, such as collagen type I (COL-I), and inhibit osteoclast-mediated bone resorption, thereby reducing connective tissue damage and promoting bone regeneration, with fascinating biological application value [24, 25]. Modulating the oxidative stress microenvironment in periodontal bone defects is crucial for achieving periodontal bone regeneration. However, the capacity of Mino to scavenge ROS is limited and insufficient to effectively manage the immunoinflammatory microenvironment associated with periodontal bone defects. Natural-derived polyphenolic compounds have excellent antioxidative and anti-inflammatory functions [26]. In addition, the rich phenolic hydroxyl structures provide a wealth of intermolecular interactions for polyphenols (PO). These two advantages lay the foundation for the development of new barrier membranes based on PO. Additionally, new barrier membranes giving spontaneous and nondependent drug release under unsuitable conditions could potentially increase the risk of antimicrobial resistance. Therefore, controlling the drug release process is crucial to prevent adverse side effects caused by high doses [27, 28]. The introduction of PO and the use of LBL technology have brought hope for the controlled release of drugs, so it is urgent to integrate PO and Mino for novel barrier membranes design for periodontal bone defects repair.

Hence, in this work, we employed LBL assembly technique to combine PO, 4-(bromomethyl)phenylboronic acid (4-BPBA, a commercial bifunctional linker) and Mino, constructing an intelligent antibacterial coating on collagen barrier membranes, to prepare a novel barrier membrane named PO/4-BPBA/Mino@COL (PBMC). PBMC demonstrated ROS-responsiveness and facilitated controlled Mino release. The incorporation of antibacterial agents and bioactive ingredients endowed PBMC with good antibacterial activity and biocompatibility, safeguarding periodontal ligament stem cells (PDLSCs) from oxidative stress. Both *in vitro* and *in vivo* results indicated that PBMC with outstanding properties, which could regulate the microenvironments of periodontal bone defects by scavenging ROS, reducing bacterial infection and enhancing the migration and osteogenic differentiation of PDLSCs, effectively promoting the regeneration of periodontal bone defects.

## Materials and methods

### Materials

The details of the materials used in this study can be found in the supplementary information (SI).

### Fabrication of PBMC

The PBMC was fabricated following improved methods from previous studies [29–31]. The details can be found in SI.

### Materials characterization

#### Scanning electron microscope

The surface morphologies of the PBMC (smooth and rough surfaces) were examined by scanning electron microscope (SEM) of FEI Quanta 250 (accelerating voltage 10 kV), which applied backscattered electrons mode (2.5 nm, 30 kV).

#### X-ray photoelectron spectroscopy

The surface chemistry of PBMC (rough surfaces) was analyzed using X-ray photoelectron spectroscopy (XPS) with an ESCALAB 250XI spectrometer, which is equipped with an AlK X-ray source (1486.6 eV) and a hemispherical analyzer (PHOIBOS 150). The survey scan was conducted at a pass energy of 100 eV with an energy step of 1.0 eV. High-resolution spectra for N 1 s and B 1 s were measured at 30 eV pass energy with the energy step of 0.1 eV, and XPS peak software (version 4.1) was used to analyze the data.

#### Fourier transform infrared spectroscopy

The Fourier transform infrared spectroscopy (FTIR) spectra of PBMC and COL were analyzed in attenuated reflection mode using an infrared spectrometer in the range of 4000–400 cm^−1^ with 32 sweeps and 4 cm^−1^ resolution.

#### Water contact angle

The hydrophilic and hydrophobic of the coatings were measured by water contact angle (WCA) via a data-physics OCA 25 and recorded with the DROP image advanced v 2.8 software under ambient conditions (25°C). The water droplets of 8 μl were deposited on the substrates and the contact angles were measured within 5 s, with the measurements repeated three times at different sites.

#### In vitro degradation assay

H_2_O_2_, as a major ROS, was used in this study to simulate the ROS-rich microenvironment at the initial stage of periodontitis [32]. Samples were soaked in 3 ml PBS, with or without H_2_O_2_, and incubated at 37°C for specific time points before removal. Following prescribed periods, the specimens were rinsed multiple times to eliminate residual salts and fully dried. Mass changes were determined by weighing the samples (*n* = 3). The remaining mass (R_M_) was calculated based on the following formula:

$$R_{M}=\left(M_{t}/M_{0}\right)\times 100\%$$

where M_0_ represented the initial mass of samples, and M_t_ represented the mass of samples after being incubated for different times.

#### ROS-responsive and Mino release of PBMC in vitro

The ROS-responsiveness and Mino release of PBMC (10 × 5 mm) were assessed *in vitro.* Incubation occurred at 37°C in 5 ml PBS, with or without H_2_O_2_. At specific intervals, 200 μl of the solution was precisely removed and placed into a 96-well plate, followed by the addition of an equal volume of fresh solution. Mino release was quantified by measuring absorbance at 375 nm, with optical density (OD) values converted to concentrations using a standard curve (supplementary Figure S2) [25]. The cumulative Mino release profiles were calculated. Experiments were conducted in triplicate.

#### ROS-scavenging assay of PBMC

The antioxidative capacity of PBMC was assessed using 1,1-diphenyl-2-picrylhydrazine (DPPH) radicals scavenging assays. A 0.1 mM DPPH solution was prepared in ethanol and kept in the dark. EGCG@COL (EC), EGCG/4-BPBA@COL (EBC), EGCG/4-BPBA/Mino@COL (EBMC), OPC@COL (OC), OPC/4-BPBA@COL (OBC), and OPC/4-BPBA/Mino@COL (OBMC) samples were added to 3 ml of DPPH solution, with a DPPH solution without samples serving as the blank control. After 30 min and 24 h dark incubation, 100 µl of the mixture was taken for OD measurement at 517 nm with a microplate reader. Each group comprised three samples. The formulation below was used to calculate free radical scavenging activity:

$$\text{Scavenging}\;\text{activity}=\left(A_{\text{control}}\;–{\;A}_{\text{sample}}\right)\;/{\;A}_{\text{control}}\times 100\%\;$$

where A_sample_ referred to the absorbance of the sample incubated in DPPH solution and A_control_ referred to the absorbance of a pure DPPH solution.

### Antibacterial assay *in vitro*

A series of antibacterial experiments were conducted to test the antibacterial effects of PBMC on four different bacteria, including *Porphyromonas gingivalis*, *Aggregatibacter actinomycetemcomitans*, *Staphylococcus aureus* and *Escherichia coli*, with a primary focus on *P.gingivalis*, which is a major pathogen in periodontitis. The details can be found in SI.

### *In vitro* cellular experiments

#### Isolation and culture of PDLSCs

The research protocol has approved by the ethics committee of Sichuan University (WCHSIRB-D-2023-400). The isolation and cultivation details of PDLSCs can be found in SI.

#### In vitro biocompatibility testing of PBMC

PBMC and COL (10 mm × 5 mm) were prepared for cell culture. COL was selected as the positive control and the culture plate served as the blank control.

For indirect toxicity, a cytotoxicity assay following the guidance of ISO 10993-6 was adopted. Briefly, the extracted solutions were obtained from the medium soaked with different materials at 37°C for 24 h. PDLSCs were seeded on a 96-well plate and incubated for 24 h. Next, the growth medium was changed to the extracts and changed every 2 days in the culture course of 5 days. After culturing for 1, 3 and 5 days, cell counting kit-8 (CCK-8) assay was used to evaluate the absorbance value at 450 nm with a microplate reader. At the same time, cell viability was detected by live/dead cell staining with a kit and visualized using an optical fluorescence microscope. For direct toxicity, after cell inoculation, PDLSCs were co-cultured with COL and PBMC. After culturing for 24 and 48 h, removing materials, CCK-8 assay was used to evaluate the absorbance value at 450 nm with a microplate reader. Cell viability was statistically calculated using the relevant formula.

To evaluate cell adhesion on the membranes, PDLSCs were cultured on the surface of PBMC and COL. After culturing for 3 days, the cell-material constructs were treated sequentially with 4% paraformaldehyde, 0.1% Triton X-100, phalloidin and 4’,6-diamidino-2-phenylindole. Then, they were imaged using a confocal laser scanning microscope (Leica, Germany).

#### Intracellular protective and antioxidative capability of PBMC

PDLSCs cells were seeded onto a 96-well plate and incubated for 24 h. The cells were then pretreated with PBMC for 24 h before being stimulated with 300 μM H_2_O_2_ at 37°C and 5% CO_2_ for 2.5 h. Additionally, a blank control group (excluding H_2_O_2_ and barrier membranes) and an H_2_O_2_ control group (only containing H_2_O_2_ but not barrier membranes) were set up. The medium was removed and the cells were washed with PBS. Next, the cell viability was detected using CCK-8 assay and the Calcein AM/PI kit. To detect intracellular ROS-scavenging levels, PDLSCs (2 × 10^4^ cells/well) were inoculated into a 48-well plate and treated with the same method as described above. Then, each well was treated with 500 μl of DCFH-DA (10 μM) followed by a 30-min incubation in the dark. Fluorescence microscopy images were collected on a Leica imaging system. For the quantitative results, the flow cytometry was conducted the fluorescence intensity.

#### Osteogenic ability effects of PBMC on the PDLSCs under oxidative stress conditions

For osteogenic induction, the cells were transitioned to the osteogenic differentiation medium (OS, growth medium supplemented with 0.1 μM dexamethasone, 10 mM β-glycerophosphate and 50 μM ascorbic acid). PDLSCs were seeded in 48-well plates and cultured for 24 h. The complete medium was replaced the OS containing 300 μM H_2_O_2_. The group co-cultured with COL, EBMC and OBMC were served as the experimental groups, cultured solely with OS served as the blank control group, and cultured with OS containing 300 μM H_2_O_2_ as the H_2_O_2_ group (*n* = 4). Part of the culture medium was replaced every 3 days. After 14 and 21 days, PDLSCs were stained with alkaline phosphatase (ALP) and alizarin red (ARS), respectively. Images of ALP staining and calcified nodules of the sample were captured using stereomicroscopy.

#### In vitro migration assay of PDLSCs

The monolayer of cells were scratched using a 200-µl pipette tip and were rinsed three times with PBS, followed by incubation at 37°C with COL, EBMC and OBMC. The images of the scratch at 0, 12, 24 and 48 h were observed under an optical microscope (*n* = 3), and the migration ratio was quantified with ImageJ software. The migration rate was calculated using the following formula:

Migration ratio (%)=(Original scratch area−Finial scratch area)/Original scratch area

#### Osteogenic differentiation

The effect of PBMC on the osteogenic differentiation of PDLSCs was detected by using ALP staining and ARS (*n* = 3). PDLSCs were cultured in OS and osteogenic differentiation conditioned medium (containing the extracts of EBMC and OBMC). After 14 and 21 days, ALP and ARS staining were, respectively, conducted, with images captured via stereomicroscopy following fixation and washing away excess dye. The ALP staining results were quantitatively analyzed using ImageJ software. To further quantify calcium mineralization, the ARS-stained samples were immersed in a 10% cetylpyridinium chloride solution at room temperature for 1 h. Finally, a microplate reader was used to read the absorbance at 550 nm.

Further determination of the expression levels of osteogenesis-related genes, including ALP, runt-related transcription factor 2 (RUNX-2), COL-I and osteocalcin (OCN), was performed using sequential quantitative reverse transcription-polymerase chain reaction (qRT-PCR). After 7 days, total RNA was extracted and reverse transcribed using the Takara assay kit. GAPDH (encodingglyceraldehyde-3-phosphate dehydrogenase) was selected as standardized gene and the data were analyzed by using the 2^–ΔΔCt^ method. The sequences of primers are provided in Table S1.

### Assessment of PBMC in periodontal bone defect model

The study protocol was approved by Sichuan University School of Stomatology’s Animal Care and Use Committee (WCHSIRB-D-2023-564). Moreover, this study utilized the resource equation method and literature-based sample size calculation to determine the required number of animal samples as 40 (*N* = 40).

Forty male SD rats (6–8 weeks old, 180–220 g) were randomly divided into five groups (*n* = 8) as follows: (i) healthy group without periodontal bone defect (healthy group), (ii) untreated group with periodontal bone defect (control group), (iii) COL group, (iv) EBMC group and (v) OBMC group.

To assess the efficacy of various barrier membranes in promoting bone regeneration under post-implant bacterial infection, a postoperative infection model was developed, adapted from previous studies [33]. Briefly, after 1 week of acclimatization, rats were anesthetized with pentobarbital sodium (2% w/v, 0.3 ml/100g), and the mucoperiosteal flaps around the maxillary first molar (M1) were raised to expose the alveolar bone. An alveolar bone defect (3 × 1.5 × 2 mm) was created on the mesial side using a dental bur under saline irrigation. The defect was covered with the sterilized barrier membranes, and the flaps were tightly sutured. Subsequently, 100 μl *P. gingivalis* (10^8^ CFU/ml) was directly injected into the implanted regions. After 4 and 6 weeks post-implantation, rats were euthanized and the maxillas were harvested. Specimens were fixed in 4% paraformaldehyde for 24 h, scanned with micro-CT (Skyscan 1174, Bruker, Belgium) at 53 kV and 810 µA to evaluate new bone formation within the defects. Following micro-CT scanning, the samples were decalcified, embedded in paraffin and tissue sections were treated with hematoxylin and eosin (H&E) staining, Masson’s trichrome staining and immunohistochemical (IHC) staining of RUNX-2 and OCN, to further evaluated the alveolar bone repair. Semi-quantitative analysis of RUNX-2 and OCN positively stained cells was conducted using Image J.

### Statistical analysis

Quantitative data were represented by means and standard deviations. GraphPad Prism 9 conducted statistical analyses. For normally distributed data, one-way Student’s *t*-tests computed *P* values for two-group comparisons, while one-way ANOVA with Tukey’s test identified significant differences among multiple groups (*P* < 0.05 denoting significance). *: *P* < 0.05, **: *P* < 0.01, ***: *P* < 0.001 and ****: *P* < 0.0001.

## Result and discussion

### Fabrication and characterizations of PBMC

**Figure 1.**
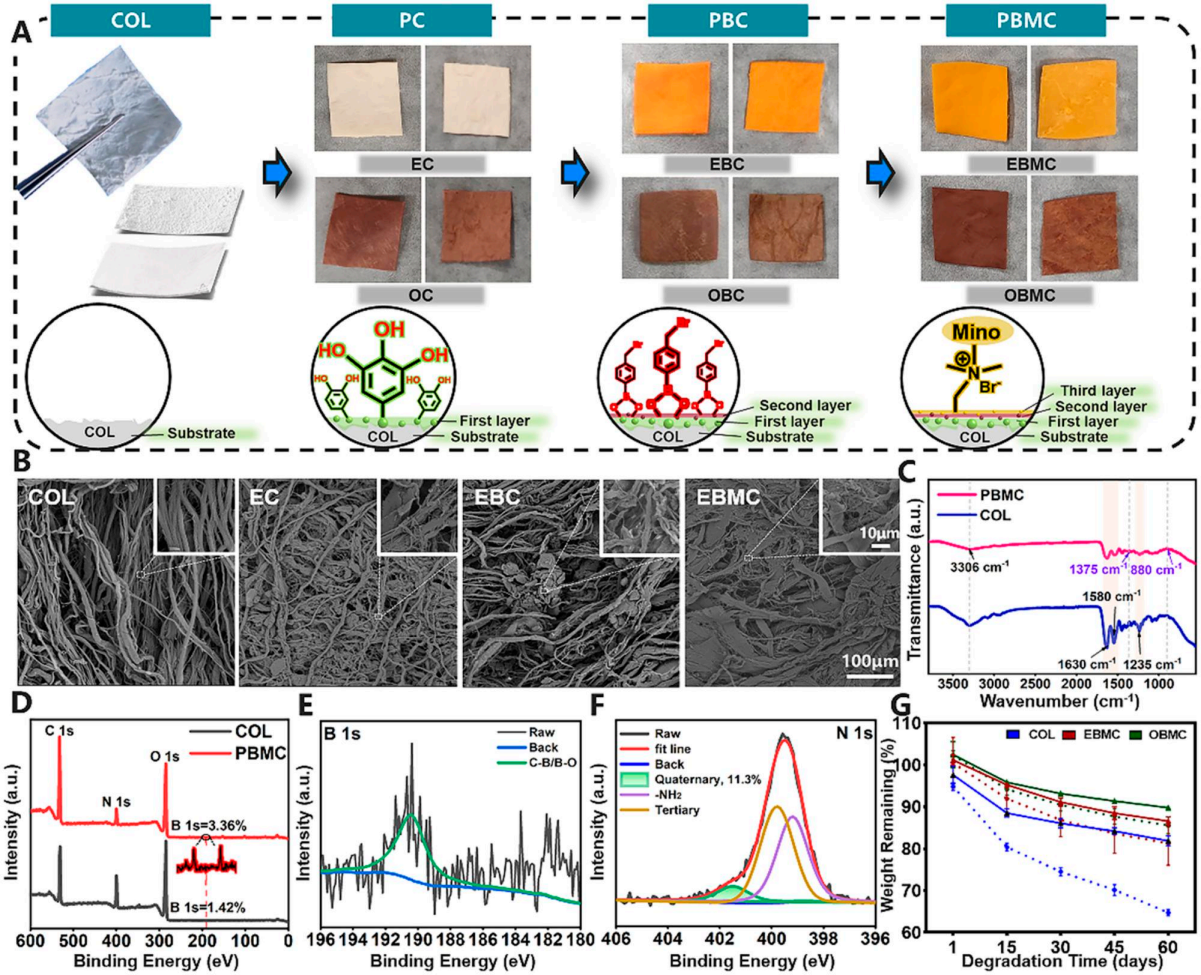
Fabrication and characterization of PBMC. (**A**) Gross appearance and schematic illustration of new barrier membranes assembled LBL with three steps. The left column of the digital photo is the smooth surface, and the right column is the rough surface (scale bar: 1 cm). (**B**) SEM images of EBMC rough surface. (**C**) FTIR of COL and PBMC. (**D**) XPS survey scan of COL and PBMC. (**E** and **F)** B 1 s and N 1 s high-resolution spectra of PBMC and (**G)** Degradation behavior of COL and PBMC with or without H_2_O_2_. The solid line represents the absence of H_2_O_2_, while the dotted line represents the presence of H_2_O_2_.

COL, widely used for periodontal bone defect treatment, features a double-layer structure where the smooth surface serves as a barrier to soft tissue, and the rough surface can promote osteoblast adhesion near defects [16]. Yet, its limited biological activity still impedes effective regeneration [33]. The novel barrier membranes designed in this study, based on COL, were crafted through the LBL assembly of antioxidant PO (EGCG and OPC), 4-BPBA and Mino, creating intelligent coatings in a sequential manner *via* polyphenolic interactions, dynamic boronate-catechol and Menschutkin reactions [31]. Digital images of the membranes showed a sequential color change, indicating the successful formation of the coating on the COL substrate. Moreover, it was still easy to distinguish between the smooth and rough surfaces (Figure 1A). In the first step, PO underwent oxidative polymerization to create a versatile adhesive layer on the COL through strong covalent and noncovalent bonds from catechols [34]. In the second step, the 4-BPBA with phenylboronic acid and bromomethyl structures could effectively react with multiple catechol structures on the first layer to form ROS-responsive borate ester bonds, and concurrently introduce bromomethyls providing active sites for further Mino loading. Finally, Mino was incorporated as an antibacterial layer *via* the Menschutkin reaction [35–37]. SEM was employed to record the surface morphology and microstructure of different membranes. As shown in Figure 1B and supplementary Figure S1A, for the rough surface of the barrier membranes, the fiber arrangement of COL was dispersed, and the fiber surface was smooth. After three steps of LBL coating, the fiber arrangement of EBMC and OBMC became relatively tight, and many attachments could be seen on the fibers, becoming relatively rough, which may be caused by the presence of PO, 4-BPBA and Mino components and the interaction between them. The SEM results of EBMC and OBMC were similar. In addition, for the smooth surface of the barrier membranes (supplementary Figure S1B), many gaps could be seen on the COL surface, while the modified EBMC and OBMC surface gaps disappeared, and the fiber arrangement became denser, which were conducive to blocking epithelial cells and playing good barrier roles.

The SEM-observed morphological alterations prompted further chemical structure analysis *via* FTIR and XPS. A comparative analysis of the FTIR spectra between COL and PBMC was presented in Figure 1C. The amide I band at 1630 cm^−1^ marked carbonyl stretch in protein links, while amide II at 1580 cm^−1^ arised from N–H bends coupled with C–N stretches. Amide III near 1235 cm^−1^ stemmed from C–N stretch and N–H bends in amide bonds. The above three troughs remained constant for all samples, indicating that the maintaining of the triple helix conformation of COL after coating. A broad trough at 3306 cm^−1^, due to hydroxyl group stretching, was more pronounced in PBMC than in COL, indicating hydrogen bond formation between collagen and PO [29]. Additionally, new peaks at 1375 and 880 cm^−1^, corresponding to the asymmetric and symmetric vibrations of the B–O bond in the phenylboronic acid group, emerged after borate ester bond formation [37]. The XPS full spectrum (Figure 1D) results showed that the contents of B elements on the PBMC increased from 1.42% to 3.36%. The visible peaks in the B 1 s spectrum (Figure 1E) were well fitted, and the presence of C–B and B–O bonds confirmed the presence of borate ester bonds in PBMC [31, 38]. The N 1 s spectrum (Figure 1F) revealed peaks for primary, tertiary and quaternary amines, with quaternary amines constituting ∼11.30%, a result of the Menschutkin reaction between bromomethyl and tertiary amine of Mino [37].

A suitable degradation rate is vital for preserving the structural integrity of bone defect regions [39]. Here, we evaluated the degradation behaviors of COL and PBMC under H_2_O_2_-induced oxidative stress. As shown in Figure 1G, under normal conditions, COL, EBMC and OBMC gradually degraded with time, but the degradation rate of COL was significantly faster than that of EBMC and OBMC. On the 60th day, COL remained at 81.89 ± 1.20%, EBMC remained at 86.58 ± 1.05% and OBMC remained at 89.79 ± 0.32%. These may be due to the interdependence of collagen fibers in EBMC and OBMC, which began to form contact and enhanced the mechanical properties of the barrier membranes. The results were consistent with a series of experimental reports conducted by the previous Man research group [29, 40]. In the presence of H_2_O_2_, the degradation rates of EBMC and OBMC increased, likely due to ROS-responsive borate ester bond breakage, leading to accelerated drug release. On the 60th day, EBMC remained at 81.31 ± 5.22%, and OBMC remained at 86.58 ± 1.05%. The degradation rate of COL was significantly accelerated, with only 64.73 ± 0.76% left on the 60th day, suggesting traditional barrier membranes may not withstand ROS damage effectively.

**Figure 2.**
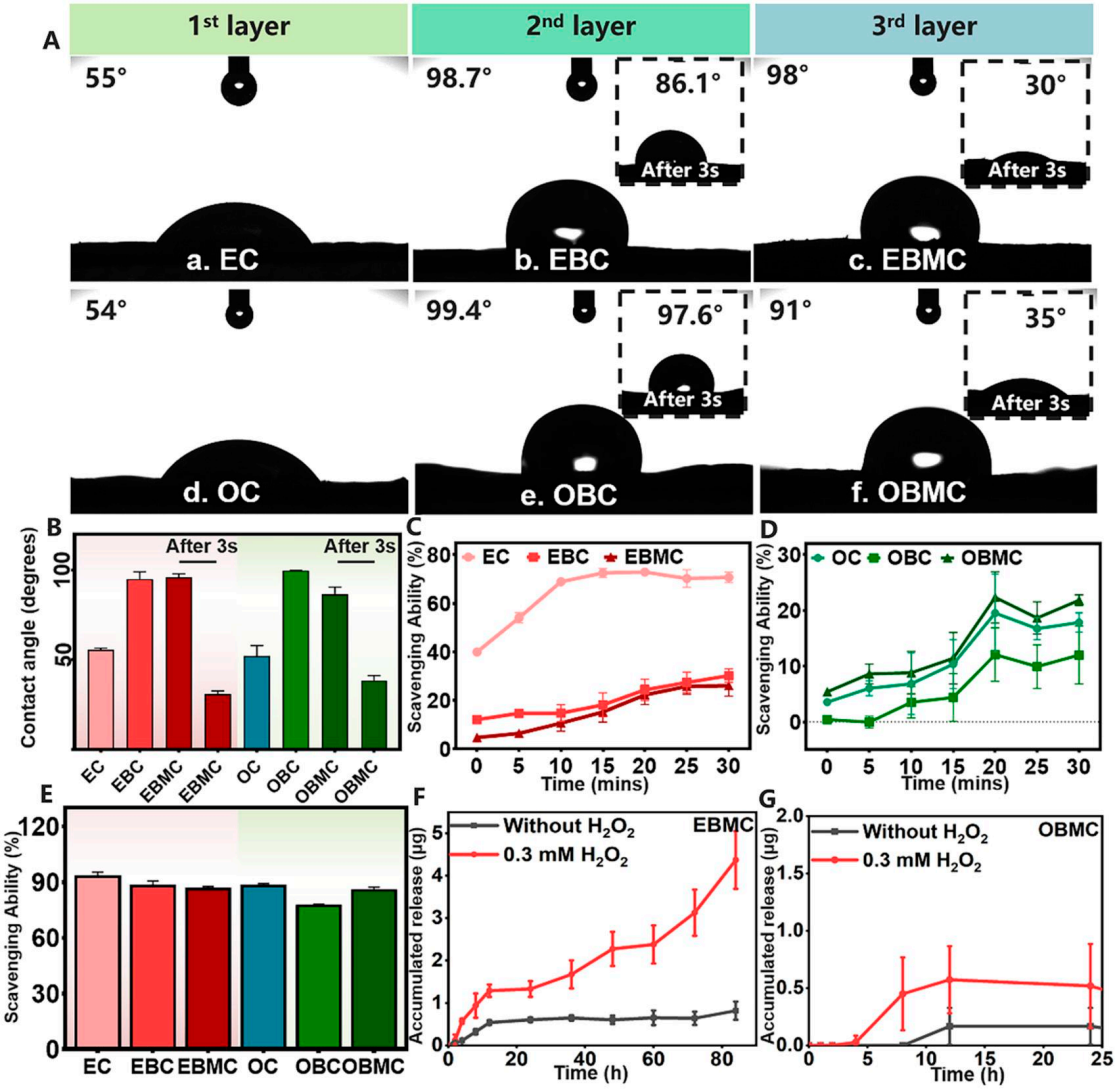
Characterization of PBMC. (**A**) Optical photos of WCA after each step of PBMC fabrication. (**B**) Quantitative results of WCA. (**C**) DPPH scavenging ability of EC, EBC and EBMC over 30 min. (**D**) DPPH scavenging ability of OC, OBC and OBMC over 30 min. (**E**) DPPH scavenging ability of EC, EBC, EBMC, OC, OBC and OBMC at 24 h. The Mino cumulative release curve and release behavior of (**F)** EBMC and (**G)** OBMC with or without H_2_O_2_.

The surface hydrophilicity and hydrophobicity of the barrier membranes fluctuated with the LBL coating process. Figure 2A and B illustrated that EGCG and OPC incorporation increased the hydrophilicity of EC and OC, likely due to their abundant phenolic hydroxyl groups [41]. The integration of the hydrophobic agent 4-BPBA decreased the hydrophilicity of EBC and OBC, indicated by the WCA exceeding 90° and minor change of WCA after 3 s, likely influenced by the hydrophobicity of bromomethyl in 4-BPBA. The bromine methyl in 4-BPBA is a hydrophobic functional group. The successful coating of 4-BPBA on PBC reduced the hydrophilicity of the barrier membrane’s surface, making it less conducive to water absorption, with minimal changes in WCA observed after 3 s [42]. The addition of Mino slightly improved the hydrophilicity of new barrier membranes, but still maintained a high WCA of over 90°. Over time, the membranes demonstrated robust water absorption with WCA dropping from the initial 98° (EBMC) and 91° (OBMC) to 30° (EBMC) and 35° (OBMC), after 3 s. Mino is a hydrophilic substance containing hydrophilic functional groups such as phenolic hydroxyl, enol hydroxyl and dimethylamino groups, which readily form hydrogen bonds with water molecules [43]. Furthermore, Chung *et al*. used 2,4,6-tris(dimethylaminomethyl)phenol as a grafting functional group to modify the surface of polyurethane, significantly enhancing its hydrophilicity [44]. Consequently, the incorporation of the Mino component altered the hydrophilicity of the PBMC surface, facilitating water absorption, and resulting in a significant change in WCA after 3 s. In the process of bone regeneration, the water absorption ability of the barrier membranes help facilitate the easy diffusion of nutrients and metabolites between cells and scaffolds [45]. Therefore, we have valid reasons to believe that PBMC have the potential to absorb exudates at the site of injury and guide cellular infiltration. Excessive ROS accumulation in periodontitis areas disrupts local homeostasis and impairs periodontal cells, and effective ROS scavenging can alter the periodontal microenvironment and facilitate periodontal tissue repair [46]. The DPPH radical scavenging assay (Figure 2C and D) was used to assess the oxidation resistance of PBMC. The assay revealed that EGCG and OPC-modified membranes possessed free radical scavenging capabilities, with EGCG being more effective. The scavenging rate decreased with each LBL layer, primarily due to fewer accessible catechol groups. The quantitative data of 24 h (Figure 2E) and digital photos (supplementary Figure S3) showed that all groups maintained strong free radical scavenging, attributed to the dynamic response of the catechol boron ester bond and subsequent polyphenolic structures [31]. These findings implied that PBMC possesses antioxidative properties beneficial for periodontal regeneration.

Regulating the oxidative stress environment within periodontal bone defects is crucial for bone regeneration and repair, and novel barrier membranes with ROS-responsive drug release behavior may be a powerful candidate for periodontal treatment [47]. Therefore, the ROS responsiveness and cumulative Mino release of EBMC and OBMC were investigated next. Figure 2F and 2G revealed a minimal baseline release under standard conditions. However, in the presence of H_2_O_2_, both EBMC and OBMC exhibited ROS-responsive release, with EBMC showing superior performance (greater release and extended duration up to 80 h). OBMC had ROS-responsiveness, but due to the low density of phenolic hydroxyl groups in OPC and the low drug loading of OBMC, Mino can only be effectively released within 24 h. Even with H_2_O_2_, the cumulative release of Mino remained low. These findings indirectly confirmed the successful LBL coating assembly on COL and suggested that PBMC with smart antibacterial coatings could respond to elevated ROS levels in periodontal defect microenvironments, offering robust antioxidant and responsive release Mino characteristics.

### Antibacterial effect of PBMC *in vitro*

Due to the lack of antibacterial function of traditional barrier membranes, early exposure and bacterial infection of postoperative barrier membranes can seriously affect periodontal bone regeneration [5, 46]. Hence, imparting antibacterial properties to traditional membranes is crucial for preventing bacterial colonization and promoting bone healing. We studied the antibacterial effects of PBMC on four different types of bacteria, with a focus on the main pathogen causing periodontitis, *P.gingivalis* [10].

**Figure 3.**
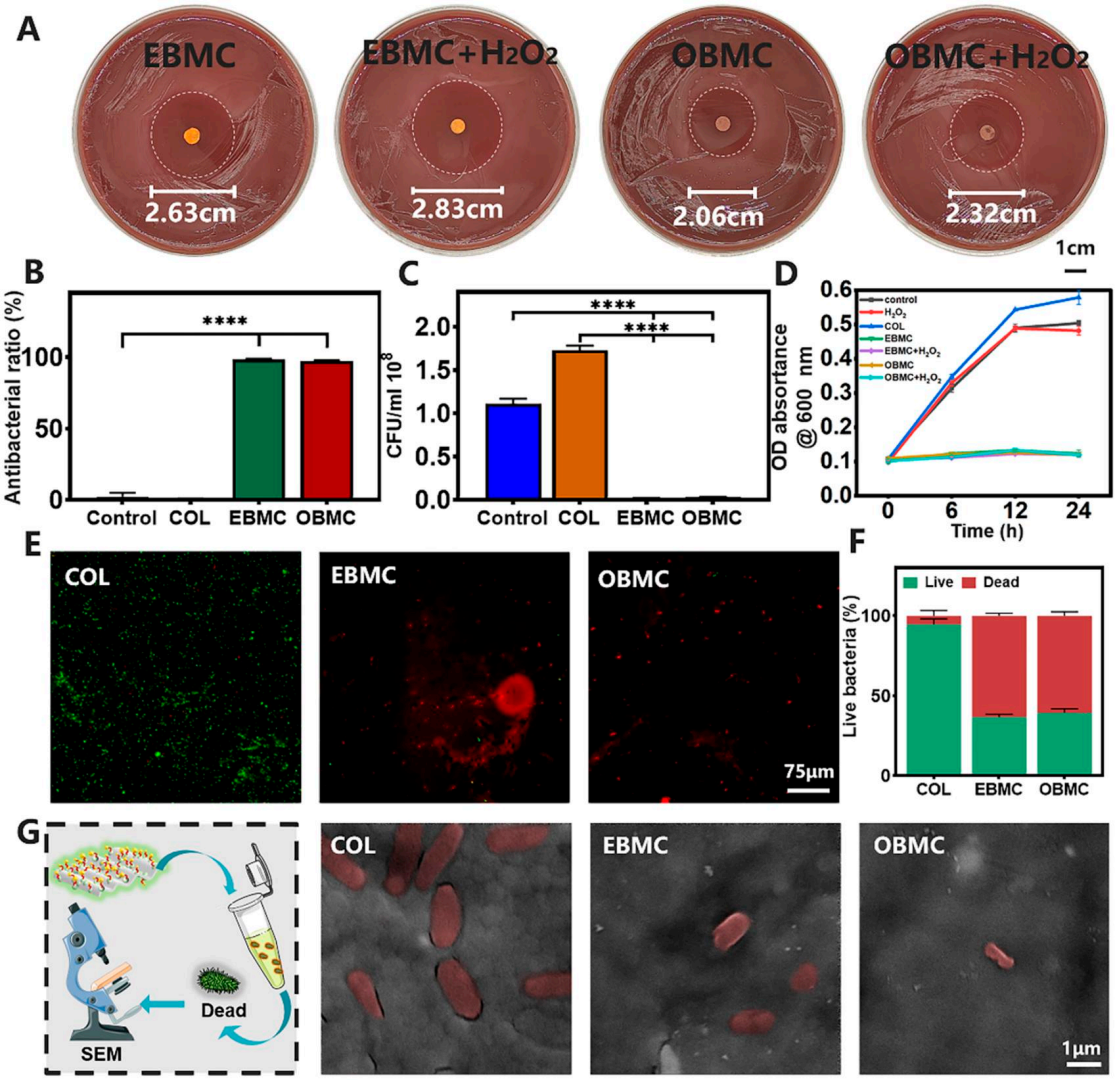
Comprehensive evaluation of the antibacterial properties of COL and PBMC. (**A**) ZOI images of EBMC and OBMC against *P.gingivalis*. (**B)** Antibacterial rate, (**C**) quantitative results of colony count and (**D**) growth curve of different samples. (**E** and **F**) Corresponding live/dead bacterial viabilities of *P.gingivalis* and fluorescence quantification results. (**G**) SEM images of the co-culture of bacteria and different samples for 24 h (****P < 0.0001).

The zone of inhibition (ZOI) of PBMC against *P.gingivalis* was first identified (Figure 3A and supplementary Figure S4). Significant inhibitory zones were observed around EBMC and OBMC, while high concentrations of bacteria were observed around the COL and H_2_O_2_ groups without any ZOI, indicating that COL without antibacterial properties and the concentration of H_2_O_2_ taken in this experiment without inhibitory effect on *P.gingivalis*. Intriguingly, under the presence of H_2_O_2_, the ZOI of EBMC and OBMC expanded, with the diameter of the EBMC inhibitory zone increasing from 2.63 to 2.83 cm, and that of the OBMC from 2.06 to 2.32 cm, which correlate with the increased Mino release in the presence of H_2_O_2_, indirectly reflecting the excellent ROS-responsiveness of PBMC. The antibacterial rates of EBMC and OBMC against *P. gingivalis* both exceeded 95% (Figure 3B). After co-culturing with *P. gingivalis* for 24 h, the residual bacterial colonies in different samples were compared. As shown in the Figure 3C and supplementary Figure S5, the COL group exhibited numerous bacterial colonies, akin to the control group and H_2_O_2_ group. Conversely, EBMC and OBMC showed significant antibacterial effects, with negligible bacterial colonies on BAP. The bacterial growth curve (Figure 3D) demonstrated that both EBMC and OBMC could effectively suppress early *P. gingivalis* growth with or without H_2_O_2_, and maintain this inhibition for up to 24 h. The growth trend of *P. gingivalis* co-cultured with COL was akin to the control group and H_2_O_2_ group, with continuous proliferation beyond 24 h, which may be attributed to COL’s lack of antibacterial properties, and providing a larger surface area for *P. gingivalis* adhesion and colonization [33].

To further certify the direct bactericidal effect of PBMC, a fluorescence live/dead assay was conducted. Figure 3E showed that COL exhibited minimal red staining and clear green fluorescence, indicative of living bacteria. In contrast, EBMC and OBMC-treated groups showed a marked reduction in green fluorescence and a significant increase in red fluorescence, confirming bacterial destruction. Quantitative fluorescence data (Figure 3F) indicated a 94.76 ± 3.39% live bacterial rate in the COL group, which dropped to 36.69 ± 1.73% and 39.39 ± 2.53% in the EBMC and OBMC groups, respectively. SEM images (Figure 3G) demonstrated *P. gingivalis* retained the complete bacterial structure with uniform, smooth and integrated surface on COL, whereas on EBMC and OBMC, the bacteria were severely damaged with compromised structures and leaked contents.

Additionally, we verified the antibacterial activity of PBMC against *S.aureus* (Gram-positive bacterium), *E. coli* (Gram-negative bacterium) and *A.actinomycetemcomitans* (another representative Gram-negative pathogen in periodontitis). As shown in supplementary Figure S6A, B, D–G, PBMC exhibited good antibacterial effects against the most common oral infection pathogens, *S.aureus* and *E.coli*, effectively inhibiting bacterial growth and proliferation during the initial colonization phase (4–6 h). *Aggregatibacter actinomycetemcomitans* is a primary pathogen responsible for invasive periodontitis [8]. As shown in supplementary Figure S6C, H and I, the control group and COL group BAP presented bacterial colonies with a grayish-white, curd-like appearance, accompanied by distinct hemolytic rings encircling the colonies. The absence of bacterial colony formation in the BAP of the EBMC and OBMC groups suggested that PBMC possessed potent antibacterial activity against *A.actinomycetemcomitans.* Both quantitative outcomes and growth curves corroborated these findings. The above results indicated that PBMC improved upon the deficiencies in antibacterial capabilities of traditional barrier membranes, showing good antibacterial effects against *P. gingivalis*, *S.aureus*, *E.coli* and *A.actinomycetemcomitans*, and can inhibit bacterial growth and proliferation in the early stages. However, it is well known that dental plaque is the initiating factor of periodontal disease, and the bacteria within the plaque exhibit a 1000-fold increase in antibiotic resistance [8]. The antibacterial effect of PBMC on plaque biofilms is not yet clear and warrants further investigation, which is necessary for better application of PBMC in periodontal treatment [48].

### Biocompatibility of PBMC *in vitro*

**Figure 4.**
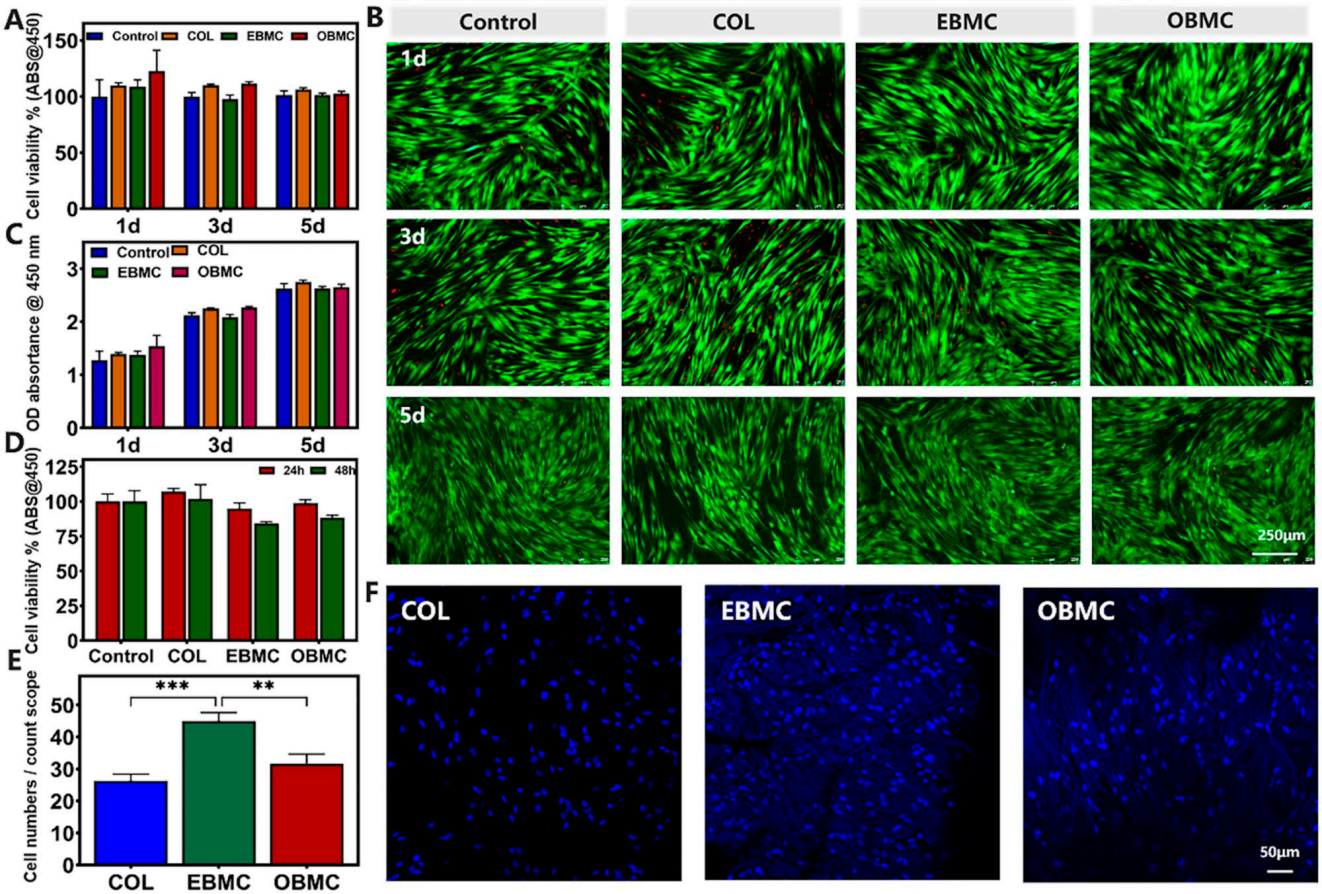
*In vitro* evaluation of the biocompatibility of PBMC. (**A**) Indirect toxicity was measured using CCK-8 assay to measure the cell activity of PDLSCs cultured with PBMC extracts on Days 1, 3 and 5. (**B**) Live/dead fluorescence staining. (**C**) Indirect toxicity was measured using CCK-8 assay to measure the proliferation of PDLSCs cultured with PBMC extracts on Days 1, 3 and 5. (**D**) Direct toxicity, EBMC and OBMC novel barrier membranes were co-cultured with PDLSCs for 24 and 48 h, and the effects of them on the cytotoxicity and proliferation of PDLSCs were detected by CCK-8 assay. (**E**) Quantitative statistics of cell adhesion. (**F**) The cell adhesion of PDLSCs on the COL, EBMC and OBMC, along with fluorescence staining of the nuclei (**P < 0.01, ***P < 0.001).

As we all know, proliferation, attachment as well as spreading occur in the first phase of cell–material interactions [49]. The results of indirect toxicity experiments showed (Figure 4A–C) that the cell viability and proliferation of PDLSCs cultured with extracts of EBMC and OBMC for 1, 3 and 5 days were similar to those of the control group and COL group. Cells proliferated continuously in all groups, and the cell populations was not significant difference between the groups. Live/dead staining showed that PDLSCs were in a good healthy state, appearing as long spindle shaped. The fields of view were mostly filled with green-fluorescent live cells. The fluorescence intensity and number of living cells in each group conformed to the laws of cell viability and proliferation. The direct toxicity results of co-culturing PDLSCs with EBMC and OBMC (Figure 4D) revealed robust cellular activity near 100% at 24 h for both groups, and a slight decrease to ∼80% was observed at 48 h, potentially attributed to the gradual release and accumulation of Mino within the membranes. Figure 4E and F indicated that on the third day, PDLSCs can adhere and proliferate well on the rough surfaces of EBMC and OBMC, and the number of cell adhesion on EBMC was higher. This may be due to the exposure of more phenolic hydroxyl groups with the degradation of PBMC and the slow release of Mino, which was beneficial for cell adhesion [34]. Due to the lower density of phenolic hydroxyl groups on OBMC compared to EBMC, the number of cell adhesion was slightly lower. The above results indicated that EBMC and OBMC exhibited good cell compatibility.

### Antioxidative capacity of PBMC *in vitro*

**Figure 5.**
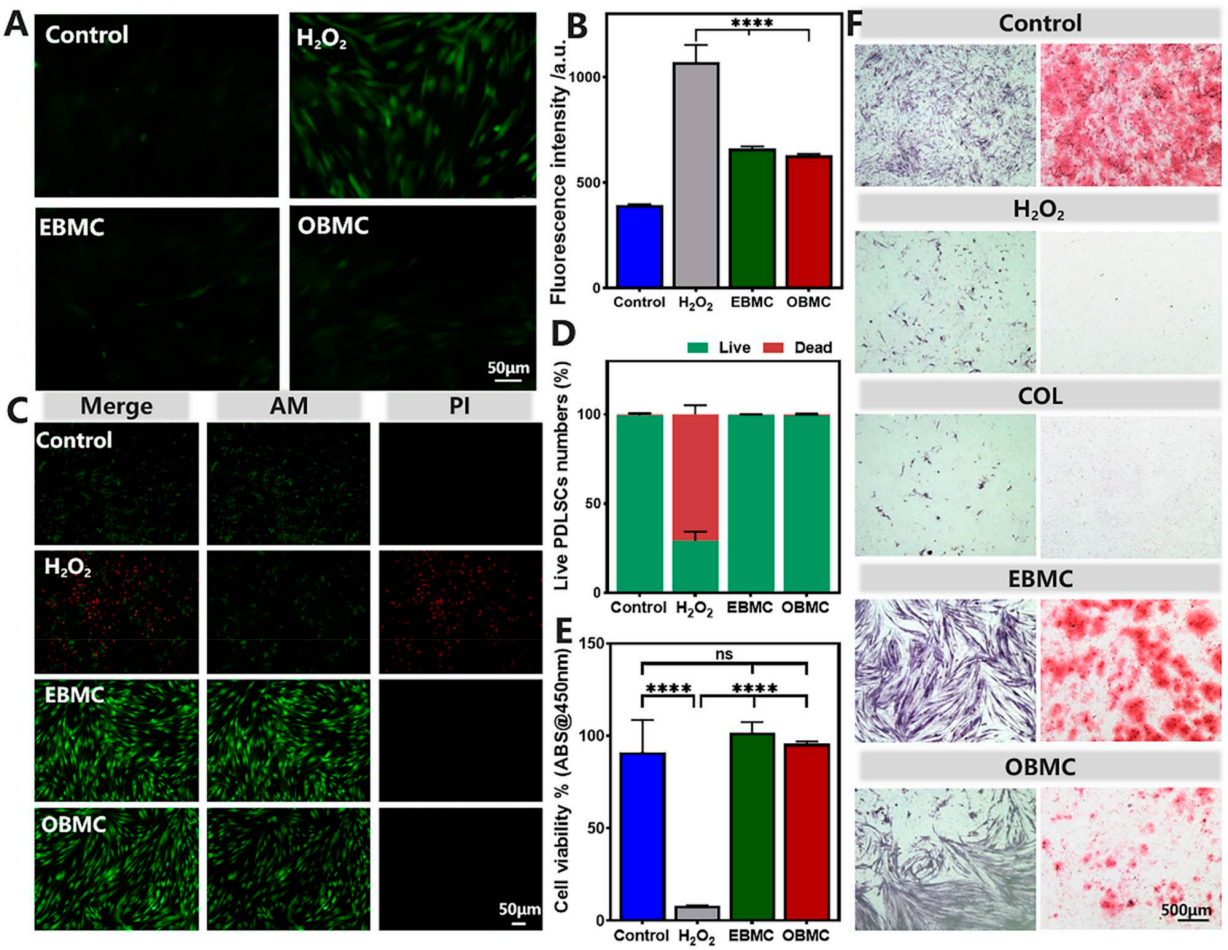
Effect of PBMC on intracellular ROS of PDLSCs cells in the presence of H_2_O_2_, including intracellular ROS-scavenging effects, cellular protective effects and osteogenic effects. (**A**) DCFH-DA staining and (**B**) fluorescent quantitative results. (**C**) Live/dead staining, (**D**) fluorescent quantitative results and (**E**) CCK-8 assay. (**F**) ALP assay (left column) on Day 14 and ARS (right column) on Day 21 under oxidative stress environment (ns: no significance, ****P < 0.0001.

PBMC has good antibacterial properties and cell compatibility. Subsequently, we evaluated its medical potential in mitigating oxidative stress and restoring cellular physiological functions in the inflammatory microenvironment. An exogenous H_2_O_2_-stimulated oxidative stress model of PDLSCs was fabricated. The intracellular ROS-scavenging ability of EBMC and OBMC under oxidative stress was evaluated using DCFH-DA staining. Representative images (Figure 5A) showed an increase ROS levels in H_2_O_2_-stimulated PDLSCs, indicated by enhanced green fluorescence. EBMC and OBMC can effectively clear H_2_O_2_-induced intracellular ROS, with fluorescence images showing no significant difference from the control group. Flow cytometry fluorescence data (Figure 5B) corroborated the DCFH-DA staining findings.

Oxidative stress occurs when endogenous antioxidants fail to equilibrate oxidative states, potentially resulting in ROS accumulation and consequent oxidative damage, which can disrupt vital cell components like DNA and proteins, leading to cell death or an inflammatory response that impairs cell activity [50]. Figure 5C illustrates an increase in red fluorescence (indicating dead cells) and a decrease in green fluorescence (indicating live cells) in the H_2_O_2_ group, along with a loss of the typical PDLSCs elongated shape. Fluorescence quantitative data (Figure 5D) conformed to the law of cell activity. CCK-8 assay results (Figure 5E) showed that the cell viability of H_2_O_2_ group was significantly reduced, while the EBMC and OBMC groups maintained high cell activity.

Next, the protective effect of PBMC on the physiological functions of PDLSCs under oxidative stress was investigated, focusing on osteogenesis (Figure 5F). Osteogenic induction was performed in an oxidative environment, revealing that H_2_O_2_ inhibited the osteogenic differentiation ability of PDLSCs, with few intact cell morphologies observed. In addition, COL was ineffective in protecting the cellular activity and functions of PDLSCs in the oxidative stress environment. As previously stated, EBMC and OBMC directly eliminated excessive ROS and enhanced the antioxidative activity of cells, thereby maintaining cellular functions. Notably, EBMC-treated PDLSCs showed increased expression of ALP (an early osteoblast marker) and mineralized nodules (a late osteoblast marker), and retained more complete cell structures. However, the ability of OBMC in maintenance cellular bone-forming is inferior, which may be associated with its lower antioxidative capacity. The aforementioned results confirmed that PBMC possesses excellent antioxidant activity, which may be attributed to the function of PO within PBMC, consistent with previous research findings [26, 51].

### The effect of PBMC on the cell migration and osteogenic differentiation

Subsequently, we detected the potential of PBMC in promoting tissue regeneration through *in vitro* migration experiments, ALP staining, ARS and qRT-PCR, laying the foundation for future *in vivo* experiments.

**Figure 6.**
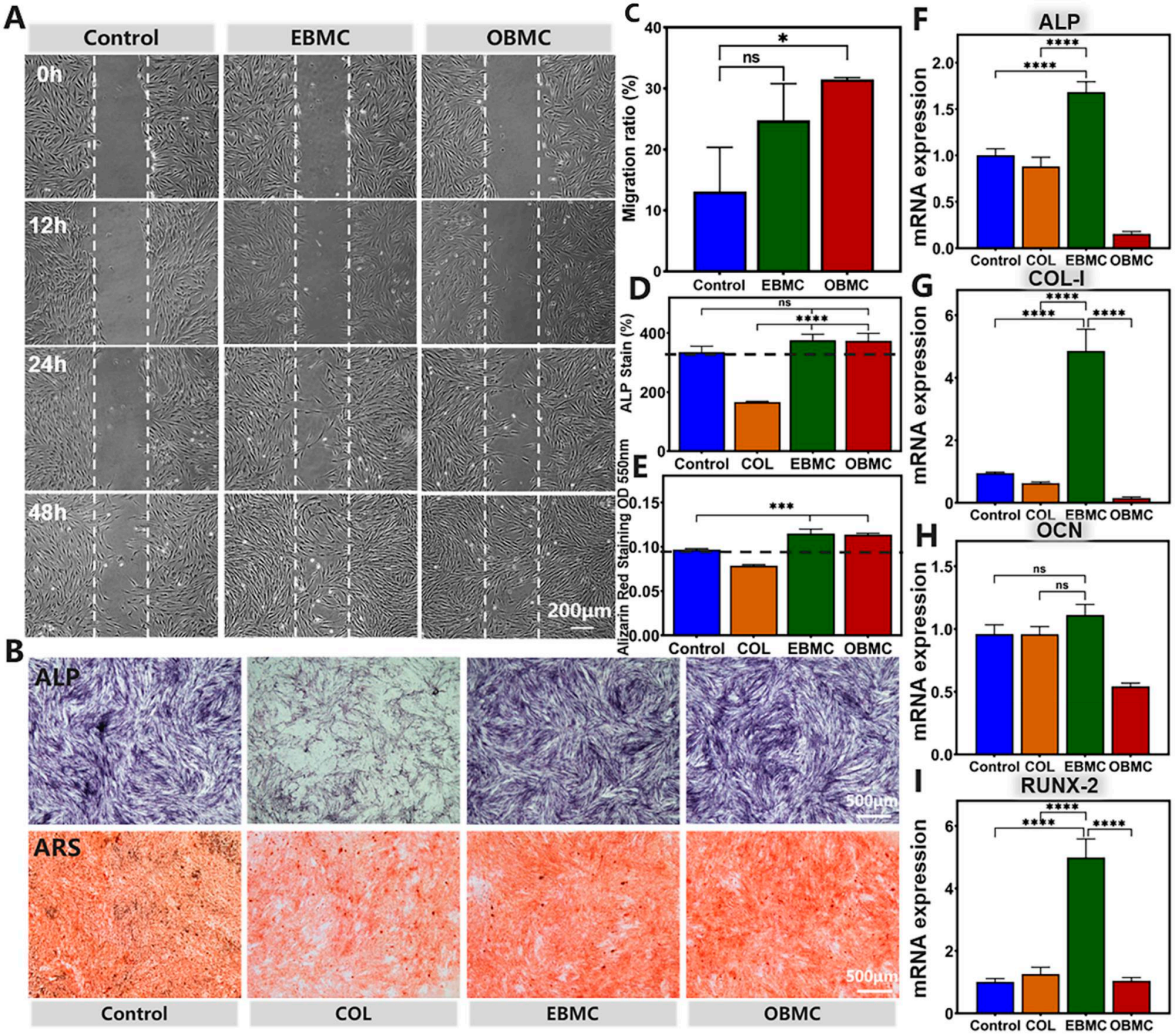
*In vitro* evaluation of the potential of PBMC in promoting tissue regeneration. (**A**) The migration of PDLSCs incubation with EBMC and OBMC for 0, 12, 24 and 48 h. (**B**) ALP staining (Day 14) and ARS (Day 21) of PDLSCs. (**C**) 24-h cell migration rate. Quantification of (**D**) ALP and (**E**) ARS, separately. (**F–I**) The relative mRNA expression of osteogenic genes (ALP, COL-I, OCN, and RUNX-2) in PDLSCs at Day 7 (ns: no significance, *P < 0.05, ***P < 0.001 and ****P < 0.0001).

Periodontal tissue regeneration encompasses the complex processes of cell proliferation, migration and differentiation [52]. An ideal periodontal tissue regeneration material must not only fend off pathogenic microorganisms and foster a conducive microenvironment, but also need to recruit stem cells in the defect area for osteogenic differentiation [49]. The cell scratch assay (Figure 6A and C) showed that EBMC and OBMC significantly hastened PDLSCs migration. The 24-h migration rates of EBMC and OBMC showed an upward trend compared to the control group, with OBMC showing a statistically significant improvement (*P* < 0.05). By 48 h, the scratch area in the EBMC and OBMC groups were almost healed, and their migration speed were faster than that of the control group. The aforementioned results may be attributed to the ability of PBMC to modulate the intracellular ROS levels in PDLSCs during *in vitro* passaging culture, maintaining a favorable redox balance within the cells, thereby sustaining better cellular activity and accelerating cell migration [53–55].

Subsequently, the impact of PBMC on the osteogenic differentiation of PDLSCs was further evaluated. ALP staining and ARS showed enhanced osteogenic capacity of PDLSCs in the EBMC and OBMC groups compared to that in the control group (Figure 6B), a trend also mirrored in the quantitative analysis (Figure 6D and E). To sum up, the EBMC and OBMC groups displayed increased ALP expression and more pronounced mineralized nodules. qRT-PCR was used to evaluate the expression of genes associated with osteogenesis, including ALP, RUNX-2, COL-I and OCN. Figure 6F–I revealed that EBMC group significantly upregulated the expression of osteogenic genes, notably ALP, COL-I and RUNX-2 by Day 7, markedly outperforming the control group, while OCN, a late-stage osteogenic marker, similarly trended upward. The above results indicated that PBMC have a favorable osteo-inductive effect on PDLSCs, which may be the result of the synergistic action of PO and Mino. First, the results from Section ‘Antioxidative capacity of PBMC *in vitro*’ of the *in vitro* antioxidant experiments indicated that PBMC can effectively eliminate ROS within PDLSCs, thereby maintaining intracellular redox homeostasis. This helped to preserve cell viability and protect cellular functions. Second, studies have shown that appropriate concentrations of PO and Mino can upregulate the expression of osteogenic-related genes COL-I, RUNX-2 and OPN mRNA, increase the activity of ALP, and enhance the mineralization of the bone matrix in PDLSCs, which were in line with the results of this study, although the exact mechanisms are not yet fully clear [52, 54, 56]. Therefore, during the extended periods of *in vitro* osteogenic induction culture, PBMC can provide a conducive environment for the osteogenic differentiation of PDLSCs, and PO and Mino can work together to exert osteogenic effects, promoting the osteogenic differentiation of PDLSCs. The osteogenic capacity of EBMC was significantly better than that of OBMC, which may be due to the superior antioxidant capabilities of EBMC, allowing for better maintenance of the redox homeostasis in PDLSCs and preventing cell apoptosis.

### *In vivo* alveolar bone regeneration assessments

**Figure 7.**
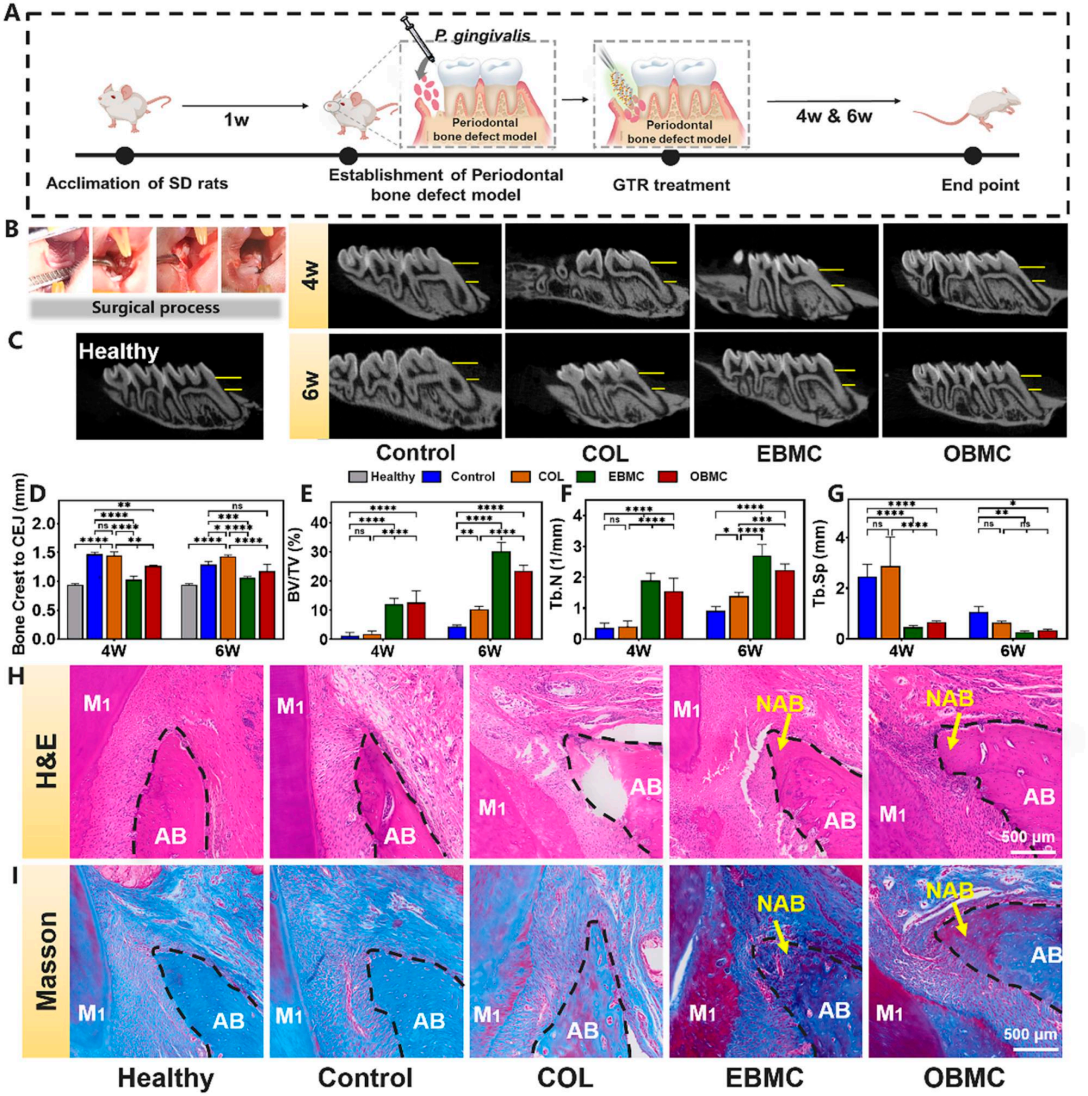
(**A**) Schematic diagram of the periodontal bone defect model. (**B**) The digital photos of surgical process: (scale bar: 1 mm). (**C**) Sectioned images of periodontal defects analyzed by Micro-CT at 4 and 6 weeks post-surgery (scale bar: 500 μm), (**D–G**) quantitative analyses of bone-related parameters including CEJ-ABC distance, BV/TV, Tb.N and Tb.Sp 4 and 6 weeks post-surgery. Histomorphological analysis of bone formation in alveolar bone defects after 6 weeks post-implantation: (**H**) H&E staining and (**I**) Masson’s trichrome staining images (AB: host alveolar bone; NAB: new alveolar bone; yellow arrows: NAB, ns represents no significance, *P < 0.05, **P < 0.01, ***P < 0.001 and ****P < 0.0001).

PBMC has excellent comprehensive performance *in vitro*, which prompts us to further access the ability to guide periodontal bone regeneration *in vivo*. We established a rat periodontal defect model, executed GTR treatment and postoperatively introduced a bacterial suspension to mimic implant infection conditions (Figure 7A). The surgical process was shown in Figure 7B. Micro-CT analysis assessed alveolar bone regeneration, determining bone repair by measuring the vertical distance between the enamel-cementum boundary (CEJ) and the alveolar bone crest (ABC) at the mesial aspect of M1 (Figure 7C). The quantitative analysis of new bone formation (Figure 7D) showed that the COL group had larger bone defects at both the fourth and sixth weeks, with the CEJ-ABC distance showing no significant change from the untreated control group. This may be due to COL’s inability to effectively address bacterial infections and high levels of ROS in the local microenvironment, thereby affecting bone regeneration at the periodontal defect site. Conversely, the EBMC and OBMC groups demonstrated a marked reduction in CEJ-ABC distance, suggesting enhanced vertical bone growth, likely related to their good antibacterial and antioxidant properties, which can counteract infections from *P.gingivalis* and modulate the local microenvironment, thereby facilitating periodontal bone regeneration [10, 57]. We also used CATn software to quantitatively analyze the regenerated alveolar bone, including bone volume/tissue volume (BV/TV), number of trabeculae (Tb.N) and trabecular separation (Tb.Sp) (Figure 7E–G). Both the BV/TV and Tb.N values for the control and COL groups were lower than those for the EBMC and OBMC groups, while the Tb.Sp values, conversely, showed an increase. Compared with the control group and COL group, the EBMC and OBMC groups showed a certain degree of improvement in the quality of regenerated alveolar bone, and the role of EBMC in promoting alveolar bone regeneration was more significant. These results collectively indicated that PBMC has a role in promoting alveolar bone regeneration. To further confirm the bone healing efficacy of PBMC in periodontal bone defect repair, we conducted histomorphological analysis 6 weeks post-implantation. The findings from the H&E and Masson’s trichrome stains are displayed in Figure 7H and I. In the healthy group, the alveolar bone exhibited a wedge shape with vascularization, surrounded by well-organized fibrous connective tissue. The control group showed few new alveolar bone (NAB) growth, largely occupied by fibrous tissue. The COL group presented limited NAB formation without discernible vessels regeneration, which may be due to the wound site being infected by *P.gingivalis*, and the bacteria were not cleaned in time, hindering the continuation of bone repair to the next stage. In contrast, the EBMC and OBMC groups demonstrated significant NAB growth within a robust blue-stained collagen matrix and new vessel formation, suggesting their potent role in promoting alveolar bone regeneration *in vivo*.

**Figure 8.**
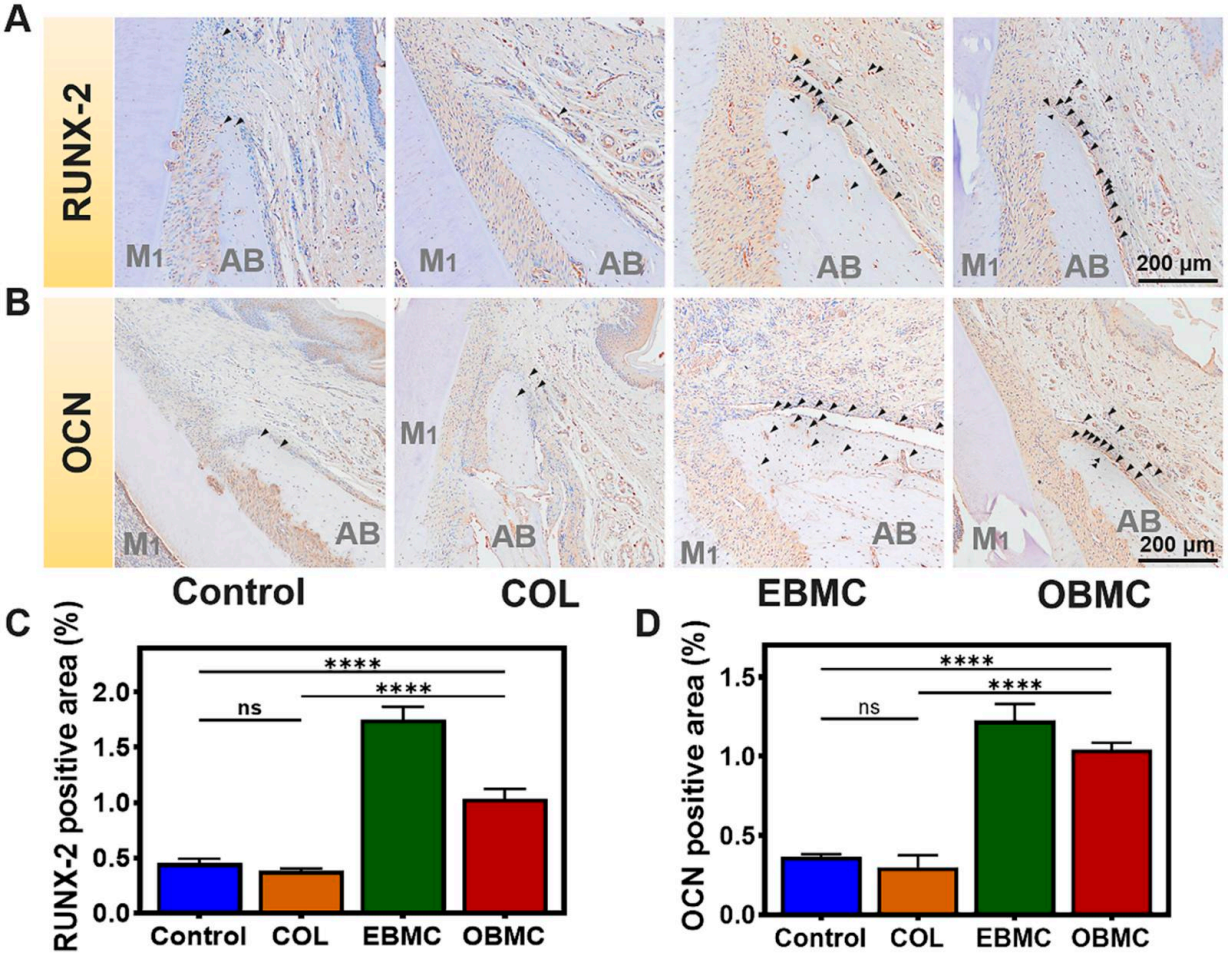
Representative IHC staining images of (**A**) RUNX-2 and (**B**) OCN in the periodontal bone defect area after 6 weeks of implantation; black arrows indicated positive cells. Quantitative analysis results of (**C**) RUNX-2 and (**D**) OCN (ns represents no significance and ****P < 0.0001).

RUNX-2 is an early osteogenic marker in the bone repair process, while OCN is a late-stage osteogenic marker [57]. As demonstrated by the IHC staining results in Figure 8A and B, 6 weeks post-surgery, there was a significant increase in the positive expression of RUNX-2 and OCN at the edges of the new bone surrounding the periodontal bone defects in both the EBMC and OBMC groups compared to the control and COL groups. The brown staining areas, indicative of positive expression, were more pronounced. The semi-quantitative results (Figure 8C and D) were consistent with the IHC staining results. The area of positive cells in the EBMC group and OBMC group was significantly larger than that in the control group and COL group, and the difference was statistically significant (*P* < 0.0001). These results further confirmed that under infected conditions, EBMC and OBMC had better osteogenic capabilities compared to COL. This is related to the good antibacterial and antioxidant effects of EBMC and OBMC, which can resist external bacterial infections while regulating the local inflammatory microenvironment, creating an excellent regenerative environment for periodontal bone regeneration. Additionally, Mino in PBMC played a role in promoting osteogenesis. Wu et al. reported in their research that at a standard therapeutic concentration of 1 mg/ml in plasma, Mino significantly increased the proliferation of human bone marrow osteoblasts without affecting their phenotype or function [60]. The Mino-loaded PLGA/PLLA membrane prepared by Liu and colleagues not only exhibited good antibacterial effects but also promoted bone regeneration, making it suitable for the repair of alveolar bone defects in progressive periodontitis [59]. Mino can facilitate the regeneration of periodontal tissue by inhibiting the release of matrix metalloproteinases (MMPs), stimulating bone formation, reducing connective tissue destruction and suppressing bone resorption [58,61].

## Conclusion

In summary, this study synthesized a novel barrier membrane with an intelligent antibacterial coating through LBL assembly, and innovatively used the commercial bifunctional linker 4-BPBA to integrate PO with Mino. PBMC has good hydrophilicity and ROS-responsiveness, which can achieve controlled release of Mino. *In vitro* experimental results indicated that PBMC has good antibacterial, antioxidative properties and biocompatibility, which can protect the cellular activity and physiological function of PDLSCs under oxidative stress conditions, and contribute to the cell migration and osteogenic differentiation of PDLSCs. The results of *in vivo* experiments showed that PBMC can still effectively promote periodontal bone defect regeneration under infection conditions. All these results indicate the potential application prospects of the novel barrier membrane PBMC in the repair and treatment of periodontal bone defects.

However, there are some properties that can be further explored and studied in the future. Exploring the antibacterial effect of PBMC on plaque biofilms is essential for its application in periodontal clinical treatment. In addition, studying the antibacterial and antioxidant effects of PBMC *in vivo* may help us investigate its mechanism of promoting bone regeneration.

## Supplementary Material

rbae058_Supplementary_Data
