# Supplementary material for: Polyphenols-based intelligent oral barrier membranes for periodontal bone defect reconstruction
Source: Regen Biomater. 2024 May 28;11:rbae058. doi: 10.1093/rb/rbae058 (PMC11157154; doi:10.1093/rb/rbae058)

**Support information**

**Polyphenols-Based Intelligent Oral Barrier Membranes** **for Periodontal Bone Defect Reconstruction**

Enni Chen^1^, Tianyou Wang^2^, Zhiyuan Sun^1^, Zhipeng Gu^2^, Shimeng Xiao^*1^, and Yi Ding^*1^

^1^ Department of Periodontics, State Key Laboratory of Oral Diseases, National Center for Stomatology, National Clinical Research Center for Oral Diseases, West China Hospital of Stomatology, Sichuan University, Chengdu 610041, Sichuan, China

^2^ College of Polymer Science and Engineering, State Key Laboratory of Polymer Materials Engineering, Sichuan University, Chengdu 610065, China

*Correspondence address. E-mail: shimengxiao817@163.com (S.M.X); yiding2000@126.com (D.Y.)

**Experimental Section**

**Material**

Bio-Gide^Ⓡ^ collagen barrier membrane (BG, Geistlich Pharma AG, Switzerland, 25 mm × 25 mm). Epigallocatechin gallate (EGCG, 98%) and oligomeric proanthocyanidins (OPC, 98%) derived from grape seed extracts were obtained from DASF Bio-Technology Co., Ltd. (Nanjing, China). Minocycline hydrochloride (Mino) was sourced from Chengdu Mendel Technology Co., Ltd. (Chengdu, China). 4-(Bromomethyl) phenylboronic acid (4-BPBA) was purchased from Energy Chemistry Co., Ltd. (Shanghai, China). H_2_O_2_ (30%) were acquired from Chengdu Jinshan Chemical Reagent Co. Ltd. (Shanghai, China). 1,1-diphenyl-2-picrylhydrazine (DPPH) was acquired from Alfa Aesar (Shanghai, China). Cell Counting Kit-8 (CCK-8), Dichloro-dihydro-fluorescein diacetate (DCFH-DA) and brain heart infusion broth (BHI) were obtained from Chengdu Baoke Biotechnology Co., Ltd. (Chengdu, China).

Alpha-minimum essential Medium (α-MEM) (Gibco) was purchased from Thermo Fisher Scientific Inc., United States. Calcein-AM/PI double staining kit was obtained from Biyun Tian Biotechnology Co., Ltd. (Shanghai, China). Tryptone, yeast extract, and agar were obtained from Oxoid (Basingstoke, UK). Ammonia (25-28 wt%) was obtained from Kelon Chemical Reagent Factory (Chengdu, China). Methanol (MeOH), and ethanol (EtOH) were obtained from Titan Technology Co., Ltd. (Shanghai, China). *Porphyromonas* *gingivalis* (*P. gingivalis*), *Staphylococcus aureus* (*S. aureus*), *Escherichia coli* (*E. coli*), and *Aggregatibacter actinomycetemcomitans* (*A. actinomycetemcomitans*) were obtained from ATCC (American type culture collection, USA). All chemicals were utilized as received, with no further purification or treatment. The deionized (DI) water employed in the experiments underwent purification using a UPH-I-10T water purification system (Chengdu, China).

**Fabrication of PBMC**

Bio-Gide^Ⓡ^ collagen barrier membranes (COL) served as substrate materials, cut to 10 mm × 10 mm, and kept dry for experiments. Briefly, EGCG and OPC, two types of PO, were each dissolved in DI water at a concentration of 0.64% (w/v), and ammonia (1 vol%) was subsequently added. COL substrate materials were immersed in these solutions and agitated (100 rpm) at room temperature for 24 h to form PO@COL (PC), including EGCG@COL (EC) and OPC@COL (OC). For the second layer, 4-BPBA (20 mg/mL) was dissolved in anhydrous ethanol containing 0.01 % volume of ammonia and coated (100 rpm) at room temperature for 24 h. The barrier membranes of coating the first layer of PO and the second layer of 4-BPBA were named as PO/4-BPBA@COL (PBC), including EGCG/4-BPBA@COL (EBC) and OPC/4-BPBA @COL (OBC). Mino was similarly coated to finalize the PBMC, including EGCG/4-BPBA/Mino@COL (EBMC) and OPC/4-BPBA/Mino@COL (OBMC) for future experiments. Each layer was cleaned and dried before the subsequent application.

**Antibacterial assay *in vitro***

The antibacterial activity of PBMC against *P. gingivalis*, a key pathogen in periodontitis, was evaluated through subsequent experiments. To evaluate bacterial growth inhibition zone, 100 μL of the bacterial suspension (10^6^ CFU/mL) was evenly spread on blood agar placeds (BAP). The EBMC, OBMC and COL (circular, 6 mm diameter) were plated at the center of BAP and incubated at 37 °C for 24 h to observe the formation of inhibition zones, with experiments conducted with and without H_2_O_2_.

The effect of PBMC on bacterial proliferation was studied by co-culturing bacteria (10^6^ CFU/mL) with different samples (n=3). The OD of the suspension was measured at 600 nm at different time (0, 6, 12 and 24 h) by using a microplate reader.

To assess the anti-planktonic bacteria ability of the PBMC, COL, EBMC and OBMC were placed in a 48-well plate，each containing 1 mL of a premade bacterial suspension (1 × 10^6^ CFU/mL). After a 24 h incubation, 100 μL of each suspension was diluted 10,000-fold, and 20 μL spread on BAP. Colony forming units (CFU) were assessed post 24h incubation at 37 °C. The antibacterial rate was calculated as:

Antibacterial ratio%=(CFU_control_-CFU_sample_)/CFU_sample_×100%

where CFU_sample_ and CFU_control_ were the number of colonies on the plate of experimental and control groups, respectively.

The effects of PBMC on bacterial viability were assessed using a live/dead BacLight kit and SEM, with fluorescence microscopy identifying live (green) and dead (red) bacteria and SEM imaging following fixation and dehydration processes.

For *S. aureus*, *E. coli*, and *A. actinomycetemcomitans*, different samples were co-cultured with prepared bacterial suspensions (*S. aureus* at 10^3^ CFU/mL, *E. coli* at 10^3^ CFU/mL, and *A. actinomycetemcomitans* at 10^6^ CFU/mL). The pure bacterial suspensions without any sample were control groups (n=3). OD values of the bacterial suspensions were measured at 600 nm using a microplate reader at various time points (0, 6, 12, and 24 h), and bacterial growth curves were plotted.

To assess the antibacterial capacities of PBMC against different planktonic bacteria, COL, EBMC, and OBMC were placed in a 48-well plate, with each well containing 1 mL of prepared bacterial suspension: *S. aureus* (10^3^ CFU/mL), *E. coli* (10^3^ CFU/mL), and *A. actinomycetemcomitans* (10^6^ CFU/mL). The pure bacterial suspensions without any sample were control groups (n=3). After 24 h incubation, 100 μL *S. aureus* and *E. coli* bacterial suspensions were diluted by a factor of 100,000, and 100 μL *A. actinomycetemcomitans* bacterial suspension was diluted by a factor of 10,000. Then, 100 μL was spread on luria-bertani agar plates or BAP, and incubated at 37°C for 24 h before counting the CFU.

**Isolation and culture of PDLSCs**

The protocol involved the collection of periodontal ligament tissues from healthy orthodontic reduced teeth or third molars from the participants aged 10-20. The tissues were processed with collagenase cultured and cultured in α-MEM with fetal bovine serum. The medium was refreshed every 3 days, and cells were passaged when 80–90 % confluence. PDLSCs from passage 3-6 were used for subsequent experiments.

**Result**

**
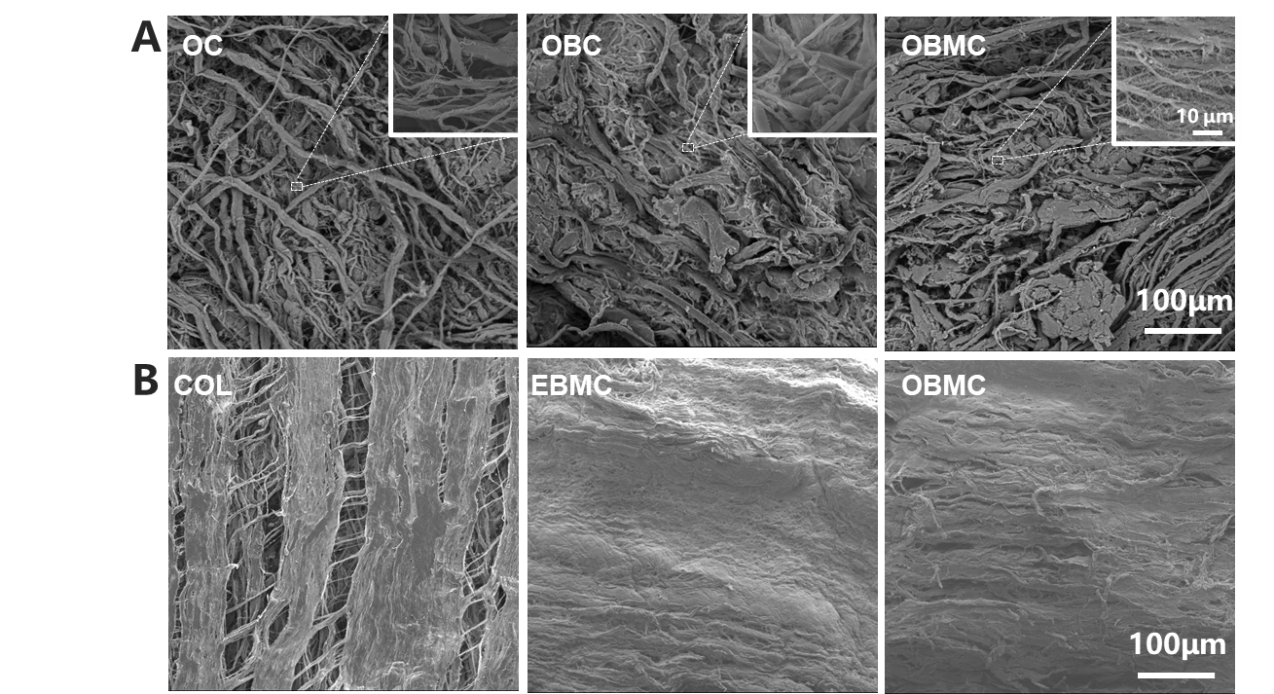
**

**Figure S1.** A) SEM images of OBMC rough surface. B) SEM images of COL and PBMC smooth surface.


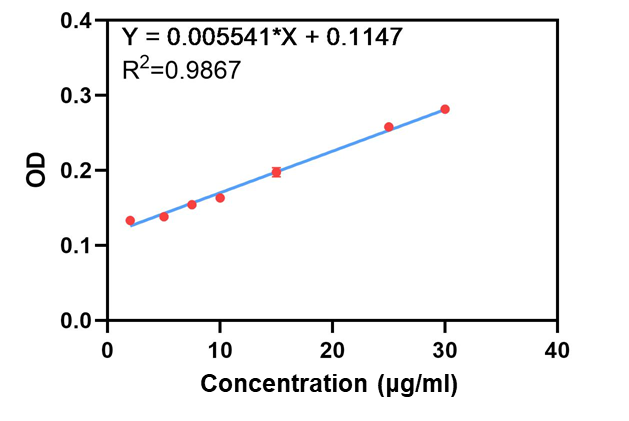


**Figure S2.** The standard curve of Mino.


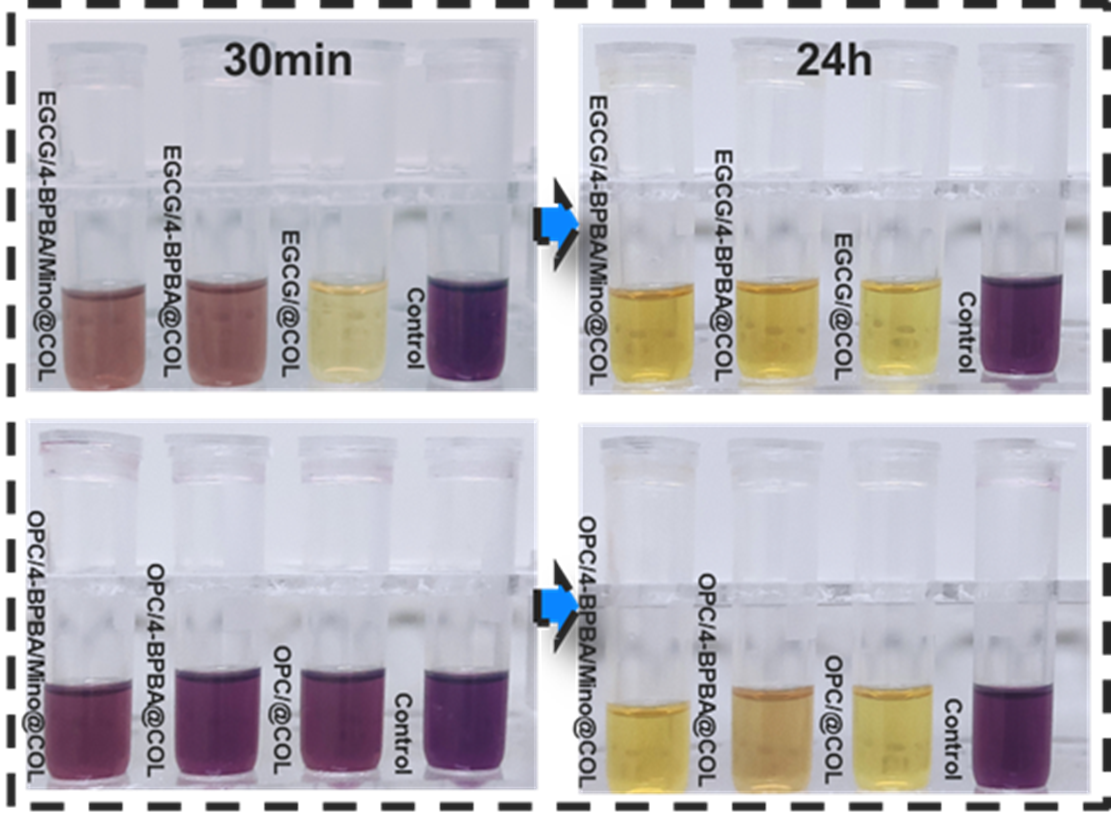


**Figure S3.** Optical photos of DPPH radical solution incubated with samples and intermediate products for 30 min and 24 h. The DPPH solution was initially purple and became yellow upon scavenging the free radicals.


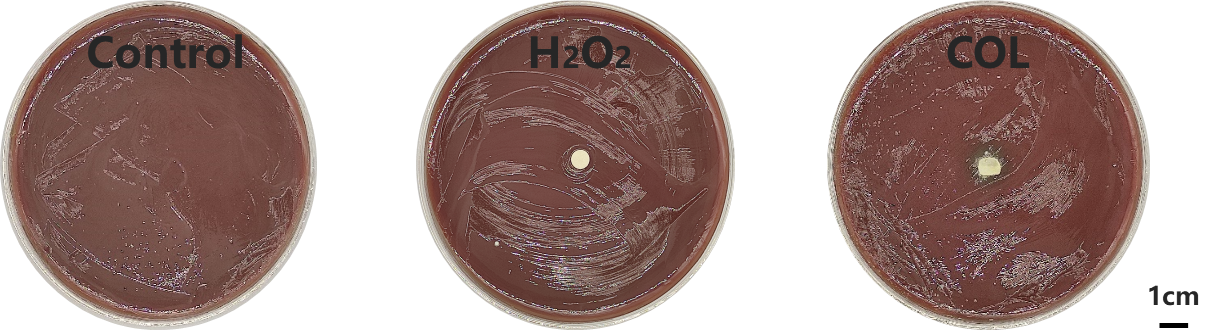


**Figure S4.** ZOI images of control, COL and H_2_O_2_ groups against *P. gingivalis*.

**
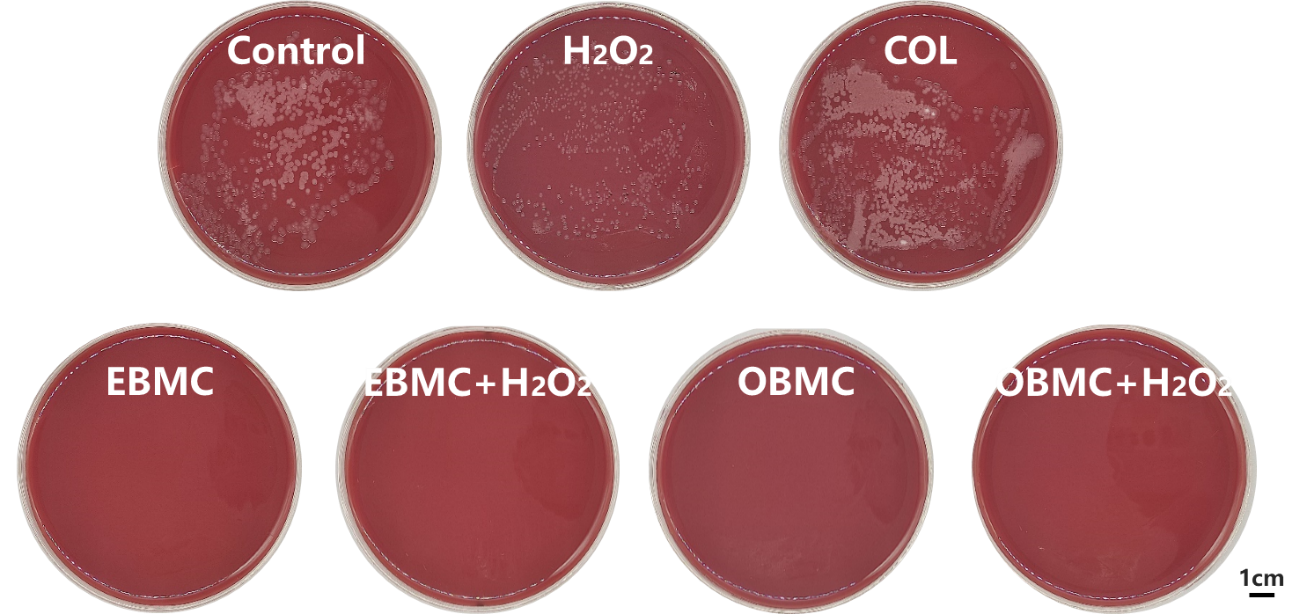
**

**Figure S5.** Bacteria colony images of COL and PBMC after different treatments, with or without H_2_O_2_.


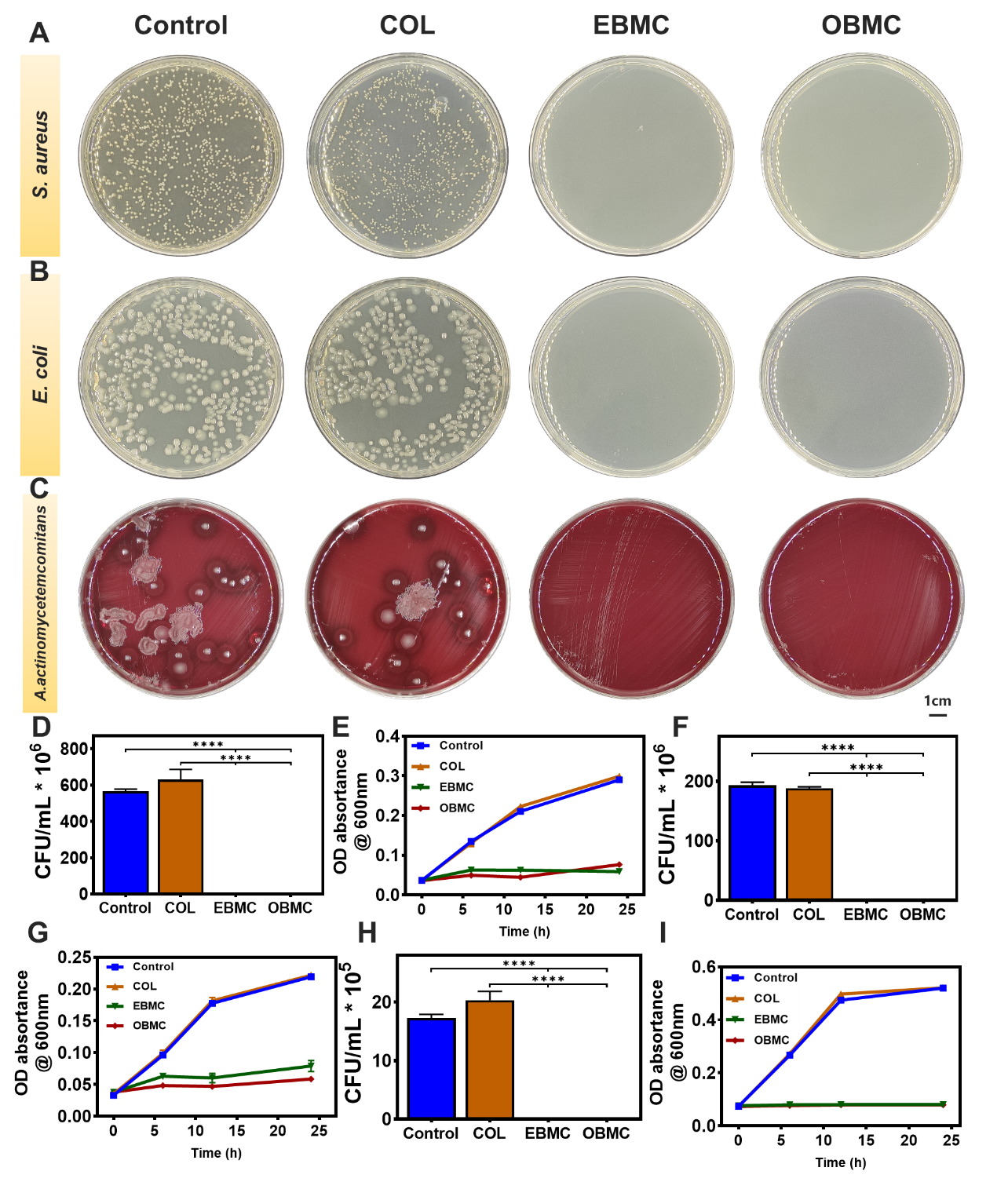


**Figure S6.** Plate colony count images of **A)** *S. aureus*, **B)** *E. coli* and **C)** *A. actinomycetemcomitans*. **D and E)** Quantitative analysis of plate colony count and bacterial growth curve of *S. aureus*. **F and G)** Quantitative analysis of plate colony count and bacterial growth curve of *E. coli*. **H and I)** Quantitative analysis of plate colony count and bacterial growth curve of *A. actinomycetemcomitans*. (****p < 0.0001 )

**Table S1.** List of primers sequences used in this study.


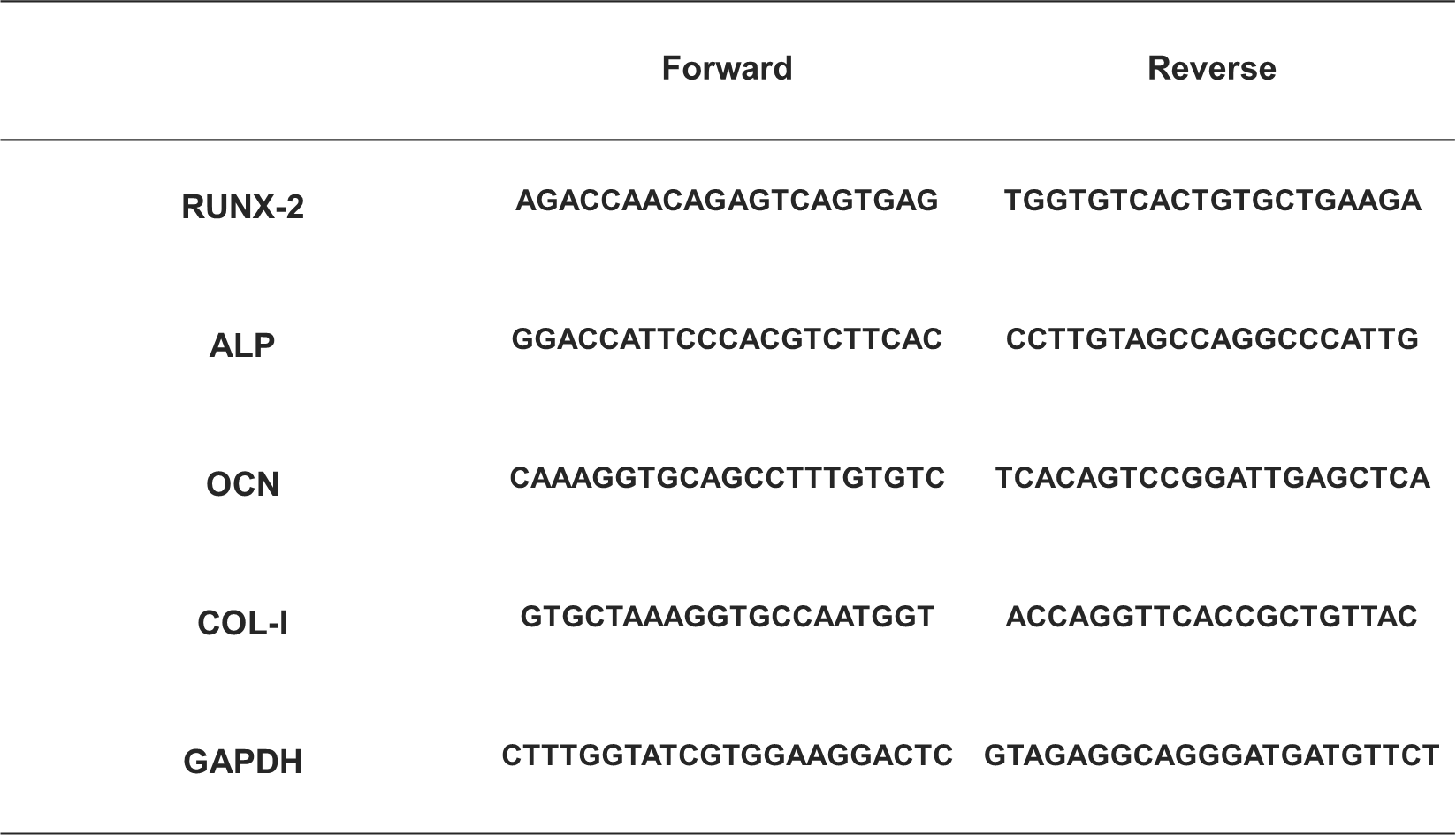

Supplement: rbae058_Supplementary_Data [file rbae058_supplementary_data.docx]
